# Utilization of Tissue Ploidy Level Variation in *de Novo* Transcriptome Assembly of *Pinus sylvestris*

**DOI:** 10.1534/g3.119.400357

**Published:** 2019-10-26

**Authors:** Dario I. Ojeda, Tiina M. Mattila, Tom Ruttink, Sonja T. Kujala, Katri Kärkkäinen, Jukka-Pekka Verta, Tanja Pyhäjärvi

**Affiliations:** * Department of Ecology and Genetics,; †† Biocenter Oulu, University of Oulu, P.O.Box 8000, FI-90014, Oulu, Finland,; † Norwegian Institute of Bioeconomy Research, 1433, Ås, Norway,; ‡ Plant Sciences Unit, Flanders Research Institute for Agriculture, Fisheries and Food (ILVO), 9090 Melle, Belgium,; § Natural Resources Institute Finland (Luke), 90570, Oulu, Finland, and; ** Organismal and Evolutionary Biology. University of Helsinki, P.O. Box 3, 00014, Helsinki, Finland

**Keywords:** Haploid tissue, megagametophyte, paralogy, *Pinus sylvestris*, allelic redundancy, short-reads, RNA-Seq

## Abstract

Compared to angiosperms, gymnosperms lag behind in the availability of assembled and annotated genomes. Most genomic analyses in gymnosperms, especially conifer tree species, rely on the use of *de novo* assembled transcriptomes. However, the level of allelic redundancy and transcript fragmentation in these assembled transcriptomes, and their effect on downstream applications have not been fully investigated. Here, we assessed three assembly strategies for short-reads data, including the utility of haploid megagametophyte tissue during *de novo* assembly as single-allele guides, for six individuals and five different tissues in *Pinus sylvestris*. We then contrasted haploid and diploid tissue genotype calls obtained from the assembled transcriptomes to evaluate the extent of paralog mapping. The use of the haploid tissue during assembly increased its completeness without reducing the number of assembled transcripts. Our results suggest that current strategies that rely on available genomic resources as guidance to minimize allelic redundancy are less effective than the application of strategies that cluster redundant assembled transcripts. The strategy yielding the lowest levels of allelic redundancy among the assembled transcriptomes assessed here was the generation of SuperTranscripts with Lace followed by CD-HIT clustering. However, we still observed some levels of heterozygosity (multiple gene fragments per transcript reflecting allelic redundancy) in this assembled transcriptome on the haploid tissue, indicating that further filtering is required before using these assemblies for downstream applications. We discuss the influence of allelic redundancy when these reference transcriptomes are used to select regions for probe design of exome capture baits and for estimation of population genetic diversity.

Coniferous trees are a dominant member of boreal forests worldwide. From an ecological, genetic, and evolutionary point of view, they represent an interesting group for comparative analyses to other seed plant groups. Due to their phylogenetic position (Li *et al.* 2015), variation in genome size, highly repetitive structure and organization rich in pseudogenes (Wan *et al.* 2018), they can serve as a useful contrasting group for comparative genomic analyses with angiosperms. However, the limited availability of genomic resources, particularly whole genome reference sequences (Birol *et al.* 2013; Nystedt *et al.* 2013; Neale *et al.* 2014, 2017; Zimin *et al.* 2014; Uddenberg *et al.* 2015; Stevens *et al.* 2016; Wan *et al.* 2018), has slowed down comparative genomic analyses in conifers with angiosperms (Chen *et al.* 2018).

Given this limited availability of whole genome reference sequences, the vast majority of current genomic analyses in conifers rely on *de novo* assembled reference transcriptomes obtained with next generation sequencing of RNA (RNA-Seq) (Li *et al.* 2015; López de Heredia and Vázquez-Poletti 2016; Baker *et al.* 2018). The use of these assembled references in conifer trees, therefore, has expanded to other applications in addition to the identification of differentially expressed genes (DEGs). These applications include comparative genomic analyses (Wachowiak *et al.* 2015; De La Torre *et al.* 2017; Baker *et al.* 2018) and expression-QTL mapping (Verta *et al.* 2016). It also includes phylogenomic analyses (Li *et al.* 2017), SNP discovery and molecular marker development (Parchman *et al.* 2010; Canales *et al.* 2014), and gene identification for exome-capture bait development (Howe *et al.* 2013; Müller *et al.* 2015). However, the generation of reliable and complete reference transcriptomes still faces several challenges. Because many non-model species are outbreeding, their genome displays high levels of heterozygosity that hampers *de novo* assembly algorithms and causes allelic redundancy (the presence of alleles of the same gene on different transcripts) and transcript fragmentation (splitting of portions of the same gene). Hence, these reference transcriptomes usually contain a larger number of contigs (transcripts) than the number of expressed genes (Gayral *et al.* 2013; Ono *et al.* 2015). Thus, downstream applications, such as the design of an exome capture, or estimation of population genetic parameters, based on these assembled transcriptomes could lead to biased estimations, and cost-ineffective experiments.

Several strategies have been employed to handle allelic redundancy and transcript fragmentation in *de novo* assembled transcriptomes (Fu *et al.* 2012; Davidson and Oshlack 2014; Davidson *et al.* 2017). These approaches include scaffolding translation mapping (STM) during the assembly (Surget-Groba and Montoya-Burgos 2010), post-scaffolding methods after assembly (TransPs) (Liu *et al.* 2014), and Orthology Guided Assembly (OGA) (Ruttink *et al.* 2013). It includes as well the identification of orthologous contigs using partial or complete genome sequences (Bao *et al.* 2013; Armero *et al.* 2017), and phylogeny-informed identification of orthologous sequences (Medlar *et al.* 2016). Although some of these strategies have been successfully applied to some crop species (*e.g.*, Stočes *et al.*, 2016), these methods rely on the quality of assembled and annotated genomic resources from closely related species to cluster allelic sequences and scaffold fragmented transcripts. In conifers, these methods have not been applied so far and allelic redundancy of assembled transcriptomes in this group is commonly reduced with CD-HIT clustering and the selection of the longest representative, with CAP3 clustering (Suren *et al.* 2016; Li *et al.* 2017), or with a combination of mapping to a reference genome and redundancy reduction with the pipeline EvidentialGene (Visser *et al.* 2018). Thus, the levels of allelic redundancy of previous published transcriptomes in conifers has not yet been determined and it is unclear to what extent allelic redundancy, and transcript fragmentation influences downstream applications. Considering the diverse range of applications of *de novo* reference transcriptomes in conifer trees (Raherison *et al.* 2012; Canales *et al.* 2014; Pinosio *et al.* 2014; De La Torre *et al.* 2015; Hu *et al.* 2016; Celedon *et al.* 2017; Porth *et al.* 2018), there is currently a need for additional strategies to assess the suitability of a transcriptome assembly for some of these diverse applications.

Paralog sequence collapse (PSC), the over-clustering of highly similar sequences leads to another type of problems in assembly and its downstream applications. These occur in the original assembly when different paralogs are assembled to create for instance mosaics (Ruttink *et al.* 2013; Yang and Smith 2014; Smith *et al.* 2015; Stočes *et al.* 2016). Allelic redundancy reduction will increase the frequency of PSC. However, most plant genomes contain several closely related gene copies (paralogs) due to whole genome or single gene duplications, making PSC and consequential paralog mapping (the mapping of reads originating from different paralogs on the same location of the reference) a common problem across species. In evolutionary and population genetic analyses, it is desirable to keep even highly similar gene copies separate, since after the duplication event they obtained distinct genomic locations, gene genealogies and evolutionary histories. Collapsing of paralogous gene copies will lead to paralog mapping and false variant calls. If paralogous sequences are collapsed into a single representative sequence in the reference during redundancy reduction, reads originating from those different gene copies will map together and variation between two paralogs will appear as polymorphism. One solution for this problem is the identification and exclusion of paralogs after the SNP calling with model based approaches or by identification of excess heterozygosity (Gayral *et al.* 2013; McKinney *et al.* 2017), which may lead to considerable loss of data in cases when PSC is common. Further, similar collapse takes place in polyploid genome assemblies, and model-based methods have also been developed to estimate ploidy level of individual contigs and variants (Margarido and Heckerman 2015; Gompert and Mock 2017). However, these methods are based on DNA reads randomly sampled from each segment and thus cannot be directly applied to RNA-derived sequence data without additional modification of the models.

Here, we take advantage of the availability of different tissue ploidy levels in conifers to determine the effects of allelic redundancy and PSC on downstream analyses. Using the single-seed haploid megagametophyte tissue in *Pinus sylvestris* L., we first tested three strategies to generate *de novo* assembled transcriptome references and second, used a novel approach to estimate levels of paralog mapping (Figure 1). PSC and the resulting paralog read mismapping in the context of this study was studied by utilizing the expected lack of heterozygosity in samples obtained from haploid megagametophyte tissues. The amount of observed haploid heterozygosity (H_o_) in various assembled transcriptomes in relation to observed and expected heterozygosity (H_E_) in diploid tissues was then used to assess the levels of PSC and its effects on estimates of genetic diversity in each assembly. We further discuss the effect of each strategy used to generate a reference transcriptome and how the levels of PSC can influence downstream applications. Particularly, we focused on the effect of PSC on reference transcriptomes for studies aiming for: 1) marker development (especially for probe design and the availability of a non-redundant reference sequence for downstream mapping analyses), and 2) use of the assembled transcriptome as a reference for population genetic analyses and genetic diversity estimates.

**Figure 1:**
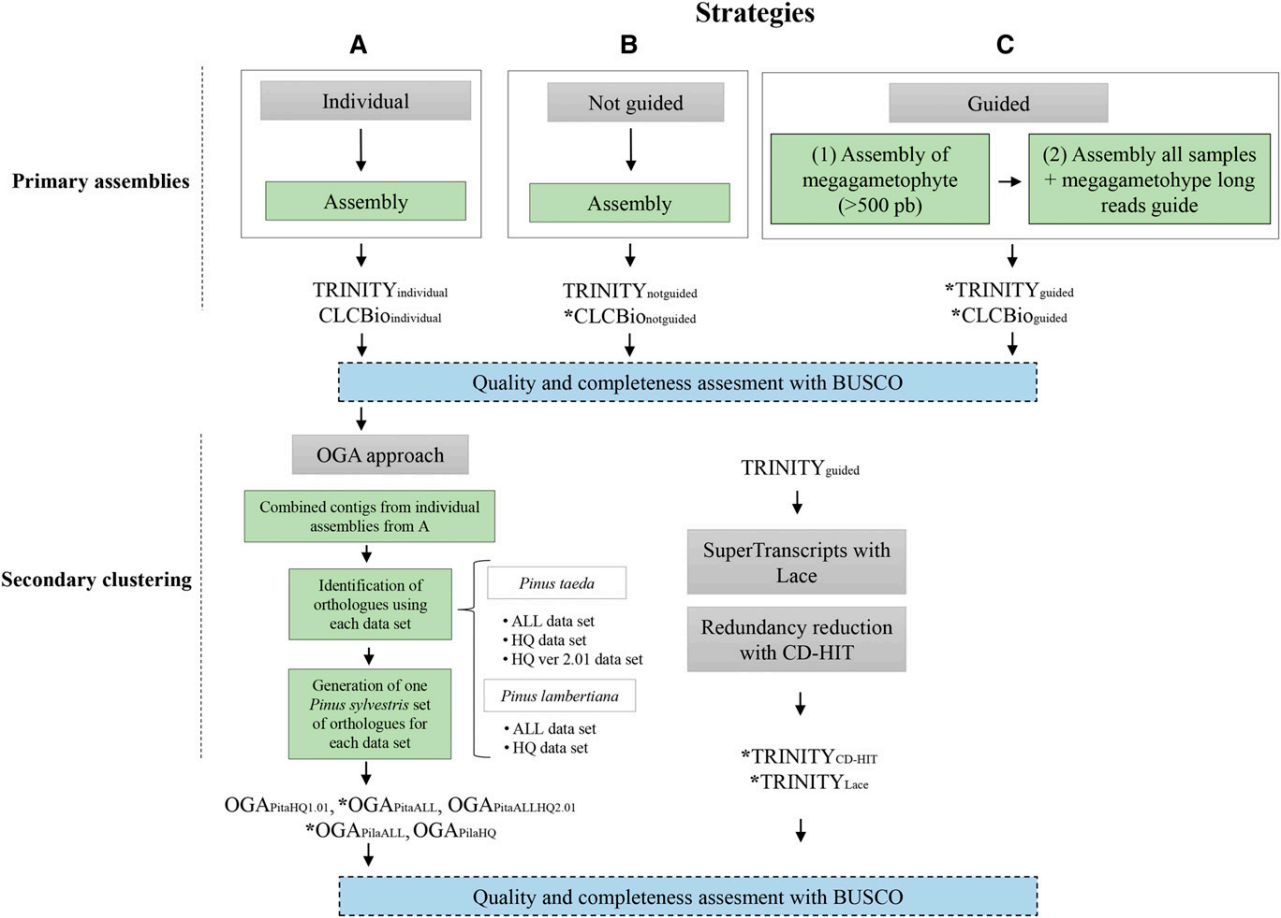
Strategies used to generate and evaluate the reference transcriptome for *P. sylvestris*. A) Individual assemblies, B) combined assembly of all reads per sample, and C) assembly of all megagametophyte (ME) per sample; retaining only > 500 bp transcripts (1) and then all different tissues per sample combined using the ME assembly as guidance sequences during the *de novo* assembly (2). Trinity and CLCbio Workbench assemblers were used on all three strategies. The secondary clustering consisted of the Orthology Guided Approach (OGA), construction of Lace SuperTranscripts followed by CD-HIT reduction of allelic redundancy. Assemblies marked with an * were assessed for levels of paralog mapping.

## Materials and Methods

### Plant material

We collected five tissues (needle, phloem, vegetative bud, embryo and megagametophyte) from six non-related individuals of *P. sylvestris* growing in a forest study site located in the Punkaharju, Southern Finland on May 26^th^ -27^th^, 2016 (Table S1). At the time of collection, male strobili were shedding pollen and the vegetative growth for the year had already started. The sampled trees grow in two close-by naturally regenerated locations (Mäkrä and Ranta-Halola, Finland). Needles (NE), phloem (PH) and vegetative buds (VB) were dissected in the field and stored immediately in RNAlater (Thermo Fisher Scientific). Samples were transported on dry ice and kept in -80° or -20° (samples in RNAlater) until RNA extraction. Megagametophytes (ME) and embryos (EM) were sampled from a single germinating seed of the abovementioned mother trees. Before germination, seeds were stored in the dark at 4°. Germination was initiated by keeping the seeds in moist paper, under constant light (300 umol/m^2^/s) and in 23° degrees for 48 hr. Each seed was carefully dissected by removing first the seed coat, the nucellar cap and layers, and taking care of separating the diploid embryo and haploid megagametophyte tissues. From each seed, megagametophyte tissue and embryo were collected, and rinsed with 70% ethanol during the dissection.

### RNA isolation, library preparation and sequencing

mRNA was directly extracted from the embryo (EM) and megagametophyte (ME) using Dynabeads mRNA Direct Micro Kit according to manufacturer’s instructions, except for a minor modification (using 200 µl of lysis buffer). Total RNA was extracted from needles (NE), vegetative buds (VB), and phloem (PH) with the Spectrum Plant Total RNA Kit (Protocol B, Sigma). After total RNA extraction, mRNA was captured using NEBNext Poly(A) mRNA Magnetic Isolation Module (New England Biolabs Inc.). mRNA extractions were treated with Turbo DNA-free Kit (Thermo Fisher Scientific). RNA concentration was quantified using Qubit 2.0 (Invitrogen) and Qubit RNA HS Assay Kit (Thermo Fisher Scientific). All libraries (6 trees × 5 tissues) were prepared using the NEBNext Ultra Directional RNA Library Prep Kit for Illumina (New England Biolabs Inc.) with a fragmentation time of 5-12 min. An insert size selection of 300 bp was targeted using a concentration of 40-45 µl per 20 µl AMPure XP (Agencourt) and between 12-15 cycles of PCR during library preparation. Libraries were indexed using NEBNext Multiplex Oligos for Illumina, Single Index Set 1. RNA library concentrations were quantified using NEBNext Library Quant Kit for Illumina and LightCycler 480 (Roche). Fragment size distributions of mRNA, total RNA and libraries were verified with 2100 Bioanalyzer RNA 6000 Pico Kit and DNA 1000 kits (Agilent). 6-12 libraries were pooled in 5 runs of an Illumina NextSeq500 instrument using pair-end 2 × 150 bp and sequenced with Mid-Output Kit (Illumina) in the Biocenter Oulu Sequencing Center.

### Strategies to generate de novo reference transcriptomes: primary assemblies

Raw read quality was analyzed with FastQC (Andrews 2010), and adapters and low quality reads were removed with Trimmomatic 0.33 (Bolger *et al.* 2014) using the following parameters: “TruSeq3-PE-2.fa:2:30:10:1:true LEADING:3 TRAILING:3 SLIDINGWINDOW:10:20”. We used three main strategies to perform primary reference assembly for *P. sylvestris* (Figure 1). The first primary assembly strategy is based on individual assemblies, using all reads per sample per genotype using CLC Genomic Workbench (www.clcbio.com) ver. 10.1 with default settings or Trinity ver.2.4.0 with default settings (with the - group-pairs-distance 1000) (Haas *et al.* 2013). This resulted in 30 separate assemblies per algorithm (here called TRINITY_individual_ and CLCbio_individual_) (strategy A in Figure 1). The second strategy first combined all reads from all samples (700 × 10^6^ reads), normalized the reads (default 50 coverage) and performed *de novo* assembly using CLCbio and TRINITY with “not guided” mode (strategy B in Figure 1, further referred to as CLCbio_notguided_ and TRINITY_notguided_). The third strategy first performed individual *de novo* assemblies, using CLCbio and TRINITY, for each of the six haploid megagametophyte tissues where only a single allele per gene is expected. We retained only transcripts > 500 bp, thus generating pseudo long reads for subsequent guided assembly. These >500 bp transcripts from the single-seed megagametophyte tissue are used for resolving isoforms, and improving assembly of complex transcripts, but they are not incorporated into the final assembly (https://github.com/trinityrnaseq/trinityrnaseq/wiki) (strategy C in Figure 1, referred to as CLCbio_guided_ and TRINITY_guided_).

### Quality assessment and completeness of primary assemblies

The assemblies obtained from the three above described strategies were evaluated in terms of several statistics, including number of contigs, N50, average contig size and number of predicted ORFs. These statistics were obtained from TrinityStats, rnaQuast (Bushmanova *et al.* 2016), and TransRate (Smith-Unna *et al.* 2016). Additionally, a metric of gene completeness of all these assemblies was determined with BUSCO ver 2.0 using the embryophyta_odb9 database (Simão *et al.* 2015) (Figure 1). We opted for these four applications in order to obtain complementary information and also to further compare similar outputs from different approaches (biological/reference based *vs.* statistical/ reference-free based metrics) (Geniza and Jaiswal 2017; Hölzer and Marz 2019). We further calculated the N50 considering only the transcripts with an expression value that represented 90% of the total expression data (E90N50). This was done for the Trinity assembled transcriptomes only (TRINITY_guided_ and TRINITY_notguided_) based on their levels of expression. First, the individual reads were separately mapped to each reference using eXpress (Roberts and Pachter 2013), then the matrix of counts was generated using the scripts of the Trinity RNAseq pipeline (Supplementary Data S1).

### Secondary clustering: the Orthology Guided Assembly (OGA) approach

After the assessment of the individual transcriptomes obtained in strategy A, we selected the reference from one assembler (CLCbio_individual_) and combined all the contigs generated from the individual assemblies. This combined set of *P. sylvestris* contigs was used for secondary assembly using OGA (Ruttink *et al.* 2013) with previously published proteomes from *Pinus taeda* and *Pinus lambertiana* (Figure 1) to guide the assembly. We either used 1) all annotated proteins (ALL) from *P. taeda* v1.0; or 2), all annotated proteins (ALL) from *P. taeda* v2.01 (Neale *et al.* 2014); or 3) all annotated proteins from *P. lambertiana* v1.0 (Gonzales-Ibeas *et al.* 2016; Stevens *et al.* 2016); or 4) only the high quality curated proteins (HQ) from *P. taeda*; or 5) from *P. lambertiana* (Table 1). Briefly, OGA first uses sequence similarity (tBLASTn e-value cut-off 1e-5 and up to 250 hits allowed) against the proteomes of the reference species to select allelic and fragmented contigs from all genotypes (assembled individually) per reference protein, then applies CAP3 clustering using default parameters for assembly (minimal overlap length of 40 bases and per cent identity of 90%) on a gene-by-gene basis (Ruttink *et al.* 2013), and finally selects the most likely orthologous CAP3 contigs per protein of the reference species. With this procedure it is possible to resolve transcript fragmentation and allelic redundancy across the individual assemblies, while generating a transcriptome reference sequence for *P. sylvestris* representing orthologs of a closely related species.

**Table 1:** Proteomes used as reference for Orthology Guided Assembly (OGA) of *P. sylvestris* transcriptomes. Number of input proteins used per reference species and the number of orthologs identified in *P. sylvestris* with the OGA approach

Species	Dataset	No. of input proteins	No. of ORFs identified	No. reference proteins with ORFS	Average length (bp	N50	N50-length	No. of orthologs in *P. sylvestris*	Reference name
*P. taeda*	ALL dataset ver. 1.01	84,525	19,123	7,008	676.90	3,367	1,158	27,241	OGA_PitaALL1.01_
	HQ dataset ver. 1.01	8,997	40,282	7,899	910.19	8,261	1,422	7,847	OGA_PitaHQ-1.01_
	ALL dataset ver 2.01	36,732	44,548	13,274	742.62	8,174	1,266	13,131	OGA_PitaALL2.01_
*P. lambertiana*	ALL dataset	85,053	9,739	3,805	807.02	1,833	1,317	22,807	OGA_PilaALL_
	HQ dataset	13,396	44,971	12,228	892.21	9,096	1,398	12,136	OGA_PilaHQ_

### Secondary clustering: Construction of SuperTranscripts with Lace and CD-HIT

Currently, there are several strategies to reduce redundant transcripts in *de novo* assembled transcriptomes for species that lack a reference genome. The generation of SuperTranscripts (representation of all isoforms by a single non-redundant contig) has been reported as an alternative for a reference genome (Davidson *et al.* 2017) and this has been implemented in the Lace software. This strategy reduces the amount of contigs by generating one single contig containing all exons arranged on different isoforms. In population genetic analyses, it is desirable to avoid the mapping of reads separately for different isoforms and information collected from different isoforms may be closer to the actual collection of exons of the gene in genomic sequence, thus the generation of SuperTranscripts with LACE it is a reasonable strategy to avoid this bias. In contrast, CD-HIT and CAP3 reduces the number of contigs based on homology percentage, and both have been reported as the most effective at decreasing redundancy in *de novo* assembled transcriptome from short reads (Yang and Smith 2013). Additional approaches include the TIGR strategy (Pertea *et al.* 2003) and the Evidential Gene pipeline (http://arthropods.eugenes.org/EvidentialGene/about/EvidentialGene_trassembly_pipe.html). Here, we opted for a combination of SuperTranscript representation followed by a reduction by CD-HIT, which in combination reduced the number of transcripts based on different perspectives. We selected the latter based on its wide application in several groups, including conifers. First, the different isoforms assembled for each gene in the TRINITY_guided_ reference were clustered into SuperTranscripts using the Lace software version 1.00 (Davidson *et al.* 2017). Briefly, we first divided the data into separate files with Lace based on cluster information from the TRINITY assembly and then generated multiple alignments for each cluster using BLAT v.35 (Kent 2002). Based on the multiple alignment of the isoforms, we applied a graph-based algorithm to generate a single sequence (SuperTranscript) containing combined information from all isoforms (Davidson *et al.* 2017). To avoid spurious isoforms with very low support, we excluded short (≤ 300 bp) and low expressed (total effective count ≤ 10 read count) isoforms prior to running Lace. In cases where all isoforms per cluster were filtered out after these exclusion criteria, the longest isoform was kept in the reference to avoid total exclusion of this sequence from the resulting reference transcriptome. We further identified SuperTranscripts that appeared to contain similar exonic sequences merged consecutively, as these kinds of “mosaics” could arise due to allelic variation and PSC. These were identified by self-blasting the SuperTranscripts. All SuperTranscripts that had blast hits within itself (other than the obvious 100% self-match) were identified as potential mosaics.

Additionally, in order to inspect the effect on commonly applied clustering procedures that are used to decrease the allelic redundancy of assembled transcriptomes, the Trinity assembly selected (TRINITY_guided_) was clustered using CD-HIT-EST version 4.7 (Li and Godzik 2006; Fu *et al.* 2012) with sequence identity cut-off (-c) 0.95, a commonly used threshold (Wachowiak *et al.* 2015; Hodgins *et al.* 2016; Li *et al.* 2017).

### Quality assessment and completeness of secondary clustering references

Finally, we evaluated the reference transcriptome assemblies obtained from the secondary clustering using the same metrics used on the primary assemblies, obtained from TrinityStats, rnaQuast (Bushmanova *et al.* 2016) and Transrate (Smith-Unna *et al.* 2016) and estimated their completeness with BUSCO using the embryophyta_odb9 database (Simão *et al.* 2015) (Figure 1). After this assessment, we selected four of the references obtained with the secondary clustering (Figure 1, references marked with an *), which were used as reference transcriptomes to map the haploid (megagametophyte) and diploid tissues for assessment of the levels of PSC.

### Assessment of the levels of PSC in references transcriptomes using haploid and diploid tissues

In order to evaluate the level of PSC on the obtained reference transcriptomes, we utilized the known ploidy level of two tissue types, the haploid (megagametophyte) and diploid tissues. In total, we employed six independent individuals, representing three different genotypes per individual: the vegetative tissues needle, phloem and vegetative bud (pooled reads of NE, PH, VB per individual) representing the diploid maternal genotype. The megagametophyte (ME) material containing a haploid maternal genome while the embryo (EM), being diploid but representing the next generation. One of the haploid genomes of the embryo is the same as the seed megagametophyte genome. The other haploid genome comes from an unknown pollinating father. Individual reads from ME, EM and NE+PH+VB of these six individuals were mapped against the seven selected reference transcriptomes obtained, four from the primary assemblies and another three obtained with the secondary clustering (Figure 1, assemblies with an *). Mapping of the reads was performed with STAR aligner (Dobin *et al.* 2013) given its suitability to allow spliced mapping of reads originating from RNA sequencing, *e.g.*, super transcriptome reference combining multiple isoforms (Dobin *et al.* 2013). We used default settings, with the exception of allowing reads to map to only one locus in the reference (–outFilterMultimapNmax 1). Otherwise, the read was considered unmapped. We also modified the default setting for filtering alignments with a mismatch of 0.025 (–outFilterMismatchNoverLmax 0.025). We used the two-pass mapping strategy (–twopassMode Basic) where STAR first performs the first pass mapping, then extracts junctions, inserts them into the genome index, and finally uses this information during the remapping of all reads in the second mapping step. Duplicates were removed with SAMtools (Li *et al.* 2009) and read groups added with the picard tool AddOrReplaceReadGroups. We generated three vcf files per reference transcriptome according to the ploidy of the tissues: one vcf for the ME (haploid), one for the EM (diploid), and one combining NE+PH+VB (diploid). Monomorphic and polymorphic sites were called with FreeBayes (Garrison and Marth 2012) using default parameters, with exceptions of using a mutation rate (-T) of 0.005, excluding indels (-i), ignoring complex events (-u), and allowing no MNPs (multi-nucleotide polymorphisms) (-X). Each vcf file with both monomorphic and polymorphic sites was filtered with vcftools (Danecek *et al.* 2011) with a minimum depth per sample set to 10, and maximum amount of missing data 0.5 per site. Further filters were applied to polymorphic sites keeping only bi-allelic SNP sites with a quality > 20. Number of heterozygous and homozygous variant calls were determined with vcftools–hardy option. Number of callable sites, sites with sufficient depth and amount of missing data per variant was determined for each transcript to allow comparable, per bp level nucleotide diversity estimates. Both observed and expected heterozygosity (π) (Tajima 1989) per nucleotide was calculated for each transcript individually. Given that the expected value of π is equal to θ (=4N_e_μ), we contrasted our observations to earlier, independent θ estimates for *P. sylvestris* (Pyhäjärvi *et al.* 2007, 2011; Kujala and Savolainen 2012; Grivet *et al.* 2017). Heterozygous calls from reads originating from haploid tissues indicate paralog mapping or sequencing/genotype calling errors and we used this to compare the level of PSC among the reference transcriptomes.

### Functional annotation and identification of contaminants on the assembled transcriptomes

The assembled transcriptomes selected as references for differential gene expression (TRINITY_guided_, see criteria later) was annotated using Trinotate, a pipeline for functional annotation of transcriptomes (Bolger *et al.* 2018). First, similarities to known proteins were detected by a BLASTX search (Camacho *et al.* 2009) (e ≤ 1e−5) against two comprehensive protein databases: Swiss-Prot (Boeckmann *et al.* 2005) and UniRef90 (The UniProt Consortium 2015) obtained from UniProt (available on Mar 8, 2018). Coding regions within transcripts were predicted using TransDecoder (http://transdecoder.github.io). The protein products identified from TransDecoder were searched for sequence similarities against the Swiss-Prot and UniRef90 protein database and for conserved protein domains using Hmmer (http://hmmer.org/) and PFam (Finn *et al.* 2014). All results were parsed by the Trinotate pipeline (https://trinotate.github.io) (Bryant *et al.* 2017), stored in a SQLite relational database, and reported as a tab-delimited transcript annotation file.

We also examined whether possible contaminants were present in the final assemblies. In order to identify contaminant sequences (not belonging to *P. sylvestris*), we performed a BLASTx search of the assembled reference (TRINITY_guided_) against Swiss-Prot limited to sequences classified as bacteria, viruses, metazoan, alveolata and/or fungi. We identified transcripts that had a BLASTx hit e ≤ 1e−5 and sequence similarity of at least 65%. Transcripts potentially originating from organelle genomes were identified by BLAST against *P. taeda* and *P. lambertiana* mitochondrial genomes and *P. sylvestris* and *P. mugo* chloroplast genomes (NCBI GenBank IDs JN854158.1 and KX833097.1) with e-value cutoff 5e-2, identity cutoff 80% and word size = 60. BLAST search of the transcripts against the *P. taeda* (v.2.01) (Zimin *et al.*, 2014) and *P. lambertiana* (v.1.0,) (Stevens *et al.* 2016) reference genome sequences was used as an additional method to identify single-copy and multi-copy transcripts. If any region of a given transcript had multiple BLAST hits with more than 85% identity, the transcript was assigned as multi-copy. We excluded 10 bp from each edge of the alignments to avoid random alignment in the edges. In addition, we required that 50% of the transcript length had sequence similarity in the corresponding reference.

### Completeness of assembled transcriptomes and comparisons with published transcriptomes of P. sylvestris

In order to determine the completeness of the transcriptome references obtained for this species, we used the core set of genes (embryophyta_odb9) dataset in BUSCO (Simão *et al.* 2015). Although this core set is only based on angiosperm taxa, it provides an estimate for the completeness of a core set of genes, also for gymnosperms. We evaluated three of the references obtained here (TRINITY_guided_, TRINITY_CD-HIT_, and TRINITY_Lace_), and compared them to previously published assemblies for *P. sylvestris*. Transcriptomes for this species have been assembled from heartwood of wounded and unwounded seedlings (Godfrey 2012), needles of two-year-old seedlings collected from five individuals (genotypes) (Wachowiak *et al.* 2015), embryos and megagametophytes at different developmental stages from a single individual (Merino *et al.* 2016). References are also available from wood cores collected from four individual mature trees (35 to 46-years-old) (Lim *et al.* 2016; Lim 2017) and pollen (Höllbacher *et al.* 2017).

### Data availability

The workflow with all the commands used is deposited in https://github.com/DI-Ojeda/Pin_syl_transcriptome. Raw reads were deposited in the NCBI SRA repository, under BioProject PRJNA531617 (SRR8996768-SRR8996761). Assemblies obtained from both programs using the three strategies (A, B and C), the set of *P. sylvestris* orthologs identified with the OGA approach with all five data sets, and the assemblies obtained using the two additional secondary clustering strategies are deposited in the NCBI TSA repository, under BioProject PRJNA531617. Files S1-S8 contains the supplementary figures and files. Tables S1-S6 contain the supplementary tables, supplemental material available at Figshare: http://doi:10.6084/m9.figshare.7623746.

## Results

### Primary assemblies

Individual assemblies (Figure 1, strategy A) obtained with the CLCbio Genomic Workbench (CLCbio_individual_) contained overall less contigs (average 84,181) than TRINITY_individual_ assemblies (average 149,943) (Table S2) and displayed slightly (on average 5%) lower BUSCO completeness scores (Figures S1 and S2). We obtained similar results in terms of average contig size, longest assembled contig, and N50 among the four assemblies obtained with the pooled assembly strategies (Figure 1, strategies B and C) with Trinity and CLCbio (Table S3). A higher number of contigs was obtained with Trinity regardless if strategy B or C was used, and this was also reflected in the number of predicted ORFs. Using the megagametophyte > 500 bp contigs (Figure 1, strategy C) as a guide during assembly increased the total number of contigs obtained in the second assembly on both algorithms. A large proportion of transcripts assembled with Trinity have low expression values. For instance, we obtained 52,208 genes with a >10 TPM level of expression for the TRINITY_guided_ assembly (Figure S4). We obtained a slightly larger number of genes with the TRINITY_notguided_ strategy (64,986 genes, E90N50 = 2,158, the N50 value obtained with only a level of expression of 90%) than with the TRINITY_guided_ strategy (50,760 genes, E90N50 = 2,388) when a cut-off expression value of >90% was used (Figure S5). BUSCO completeness scores were also higher on these latter assemblies compared with strategy B. The highest BUSCO completeness scores and the lowest number of fragmented transcripts was obtained with the TRINITY_guided_ assembly (Figure S6 A).

### Secondary clustering: Orthology Guided Assembly (OGA)

We used the individual assemblies obtained with the CLCbio (CLCbio_individual_), which contained overall considerably lower complexity (measured by the number of unique contigs per sample, as function of the total number of reads available) (Figure S3). Given its reduced size compared to TRINITY assemblies, the identification of orthologs with the proteomes of *P. lambertiana* and *P. taeda* required less computational time. We combined the individual transcripts for each of the 30 samples resulting in a total of 2,495,509 transcripts (average length = 642.76 bp, N50-length = 930 bp) and used them as an input for the identification of orthologs with the OGA approach. Overall, we obtained a higher number of orthologs of *P. sylvestris* using the ALL proteome datasets for both reference species, with higher numbers with *P. taeda* (27,241 orthologs) than *P. lambertiana* (22,807 orthologs) (Table 1 and S5). This was also reflected in the BUSCO completeness scores, where a more complete core gene set was recovered with the ALL proteome datasets for *P. taeda* (42.6%) than for *P. lambertiana* (42.0%) (Table S4 and Figure S6B). The ability to recover a full-length *P. sylvestris* transcript per gene with the OGA approach was determined by plotting the distribution of the fraction of the assembled transcript length *vs.* the respective *P. taeda* or *P. lambertiana* reference sequence length. We found that the majority of the transcripts obtained using the five reference proteomes (Table 1) were fragmented (broken or partially assembled transcripts) (Figure S7). For instance, using the latest version of the *P. taeda* protein set (Table 1, ALL dataset ver. 2.01) resulted in the recovery of 13,131 *P. sylvestris* transcripts (out of the 36,732 *P. taeda* reference proteins), but only one-third of the *P. sylvestris* transcripts encodes a full-length protein with a similar length as the *P. taeda* reference protein (ratio close to 1) (Figure S7).

### Secondary clustering: Reduction of allelic redundancy with Lace and CD-HIT

After the previous assessments, the TRINITY_guided_ reference was further analyzed to reduce its redundancy with the construction of SuperTranscrips (Lace) and with CD-HIT. From the original set of 1,307,499 contigs in the TRINITY_guided_ assembly, a total of 787,820 SuperTranscrips were obtained with Lace, of which 71,344 (9%) included multiple isoforms. Out of the multi-isoform clusters, most had a few (<5) isoforms (Table S5). With CD-HIT, we further reduced the number of transcripts by 9%, resulting in a set of 717,762 transcripts.

### Assessment of levels of paralog sequence collapse (PSC)

To evaluate the level of paralog read mapping due to PSC on the reference transcriptomes obtained, patterns of heterozygosity among haploid and diploid tissues were compared on the seven selected reference transcriptomes (Figure 1). The summary of SNP and genotype calling results is presented in Table 2. It is noteworthy that both the size of the transcriptome and the number of callable sites (monomorphic and polymorphic sites passing the filters) are very different among assemblies, the latter varying from 6.7 × 10^6^ callable sites in OGA_PilaALL_ to 102 × 10^6^ callable sites in the TRINITY_CD-HIT_. Larger references resulted in lower average depth and vice versa, because smaller assemblies force the same amount of reads to map to smaller number of locations. In addition, the number of callable sites will act as a denominator in per nucleotide estimates, thus reducing the apparent level of diversity in the reference transcriptomes with more callable sites. Therefore, for evaluating the behavior of different assemblers, we also considered the haploid/diploid ratio of H_o_ in addition to the absolute number of variants per nucleotide, as the ratio is less affected by the assembly size.

**Table 2:** Summary of the assessment of PSC levels utilizing observed heterozygosity patterns on the seven reference transcriptomes selected. NA = not analyzed. *Only including genes where there are >100 callable sites. ^1^Rough estimate for total sites from Grivet *et al.* (2017), assuming that one-third of coding sites are non-synonymous and two-third are synonymous. ^2^Assuming Hardy-Weinberg equilibrium

Assembly	Total assembled bases	No. of callable sites*			Expected heterozygosity per bp at callable sites (x 1000)			Observed heterozygosity per bp at callable sites (x 1000)			Ratio of H_O_		Ratio H_o_/H_E_		
		ME	EM	NE,PH,VB	ME	EM	NE,PH,VB	ME	EM	NE,PH,VB	ME/EM	ME/NE,PH,VB	ME	EM	NE,PH,VB
TRINITY_guided_	667,499,116	14,501,929	19,472,366	NA	0.85	0.86	NA	0.29	0.86	NA	0.34	NA	0.34	1.00	NA
CLC_guided_	233,918,293	19,255,392	26,397,168	48,098,347	0.79	0.74	0.75	0.22	0.74	0.78	0.29	0.28	0.28	1.00	1.03
CLC_notguided_	207,640,702	23,803,859	28,354,915	42,051,752	0.72	0.72	0.8	0.23	0.78	0.86	0.29	0.26	0.31	1.08	1.08
TRINITY_Lace_	399,688,510	27,934,641	33,810,952	28,094,261	0.69	0.71	0.83	0.25	0.76	0.91	0.33	0.28	0.37	1.08	1.10
TRINITY_CD-HIT_	379,552,297	91,640,833	101,596,814	47,019,030	0.12	0.14	0.75	0.03	0.09	0.85	0.28	0.03	0.22	0.67	1.14
OGA_PilaALL_	16,643 820	6,748,147	7,852,153	11,128,428	0.54	0.52	0.59	0.26	0.64	0.74	0.40	0.35	0.47	1.24	1.26
OGA_PitaALL_	18,721 689	6,889,082	8,303,569	11,995,251	0.58	0.56	0.63	0.27	0.69	0.78	0.39	0.35	0.47	1.23	1.25
**Expectation**					**4.2**^1^	**4.2**	**4.2**	**0**	**4.2**	**4.2**	**0**	**0**	**0**	**1**^2^	**1**^2^

As expected, observed heterozygosity per nucleotide was lower in megagametophytes than in diploid tissues on all the assemblies assessed. However, some observed heterozygosity in haploid tissue in all assemblies suggest that all the assessed transcriptomes still result in considerable amount of false heterozygous calls, either originating from paralog read mapping or from errors during mapping and variant calling (Table 2). We found that the relative amount of false heterozygous calls (ratio of H_o_) was lowest in TRINITY_CD-HIT_ (TRINITY_guided_ assembly, followed by creation of SuperTranscripts and clustering redundant contigs with CD-HIT, Table 2). In contrast, haploid *vs.* diploid observed H_O_ ratio was the highest among all assemblies in OGA assemblies. Also, the H_o_/H_E_ ratio was considerably higher than one for both OGA assemblies in diploid tissues suggesting excess heterozygote calls likely due to PSC.

As a further validation of our methods of identifying levels of paralog read mapping based on haploid heterozygosity, we compared the heterozygosity level in single and multi-copy genes identified in the TRINITY_Lace_ (Figure 1). We expected the paralog mapping and hence haploid observed heterozygosity to be higher in multi-copy genes that are prone to PSC. In diploid genotypes, multi-copy genes had slightly higher heterozygosity than single copy genes. In haploid genotypes, the majority (93%) of genes with H_o_ > 0 were found in multi-copy genes (Figure S8). On the other hand, 30% of the multi-copy genes, but only 14% of the single-copy genes (where we obtained sufficient data for SNP calling) have H_o_ > 0. Thus, the two independent methods yield consistent but not completely overlapping methods to identify loci prone to PSC.

### BUSCO completeness and functional annotation of the assembled transcriptome

**Figure 2:**
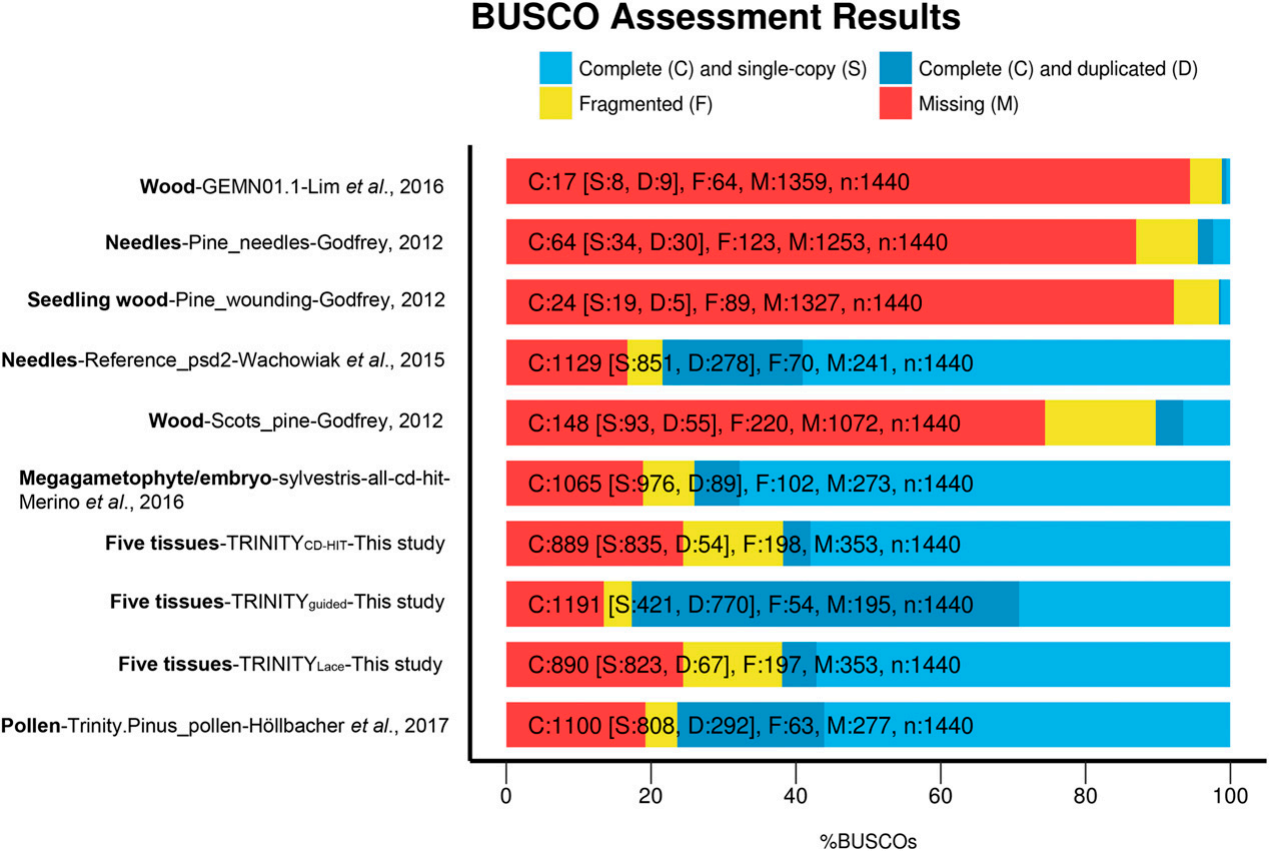
Percentage of completeness on the core set of genes in BUSCO of the assemblies obtained in this study in comparison with published transcriptomes.

Our final assemblies ranged between 61.0% and 82.7% completeness, with the highest level recovered in the TRINITY_guided_ assembly (Figure 2). In contrast to previous published assembled reference transcriptomes of *P. sylvestris* that used only one or two different tissues (Wachowiak *et al.* 2015; Lim *et al.* 2016; Merino *et al.* 2016; Höllbacher *et al.* 2017; Lim 2017), our assemblies were based on five different tissues (needle, phloem, vegetative bud, embryo and megagametophyte) from six individuals. The number of full-length genes captured in the TRINITY_guided_ assembly is also reflected in the number of ORFs predicted with TransRate, which was larger than the previously available reference for *P. sylvestris* (Table S6). The TRINITY_guided_ reference therefore represents the most complete available resource for this species to date. Using the Trinotate pipeline we annotated the 1.3 million transcripts of this assembly and found a large number (50,760) of likely full-length transcripts enriched in the E90 set (Figure S5a) and at least 64,260 complete ORFs predicted with Transdecoder. In addition, Trinotate found 24,780 genes matching Pfam Tracheophyta protein hit (Supplementary Data S1).

## Discussion

Considering the large and complex genomes of conifers, transcriptomes are increasingly being used as a reference resource in a variety of applications (Müller *et al.* 2015; Wachowiak *et al.* 2015; Suren *et al.* 2016). Particularly in Pinaceae, where the vast majority of transcriptomes have been generated (López de Heredia and Vázquez-Poletti 2016), the number of assembled contigs (transcripts) is always larger than the actual number of genes estimated based on their genome sequence (Table S6) (Nystedt *et al.* 2013; Neale *et al.* 2014; Gonzales-Ibeas *et al.* 2016). This is not a unique to conifers, but it is a common output in assembled transcriptomes of other organisms. Splitting of alleles originating from heterozygote material, pooling of multiple genotypes, contaminants and rare transcripts accumulating with increased sequence depth all have potential contribution to the increase the number of contigs in *de novo* assembled transcriptomes. On the other hand, paralogous gene sequences are erroneously collapsed into a single reference sequence, causing paralog sequence collapse (PSC). This is one of the main limitations and potential source of error is the level PSC in the reference transcriptome. There are currently several strategies to deal with redundancy, but most rely on the availability of available genomes and/or high quality annotated references.

One characteristic of conifers (and other gymnosperms) is the presence of easily accessible megagametophyte tissue, and their amenity to extract haploid genome information from it. Here we employed two popular assemblers with contrasting features (in terms of availability and output), Trinity and CLC Genomic Workbench for our primary assemblies with the purpose to provide reference to benchmarked data on *de novo* assembly. Then we employed alternative strategies to reduce paralogs with the secondary clustering. Our selection of secondary clustering strategies was based on strategies previously employed on model and non-model systems and that could be a useful comparison for *P. sylvestris* (and most gymnosperms) with limited available genomic resources. We used the variation of ploidy in *P. sylvestris* tissues to assess their utility during the *de novo* assembly and employ a novel strategy to estimate levels of PSC on the assembled transcriptomes. The same approach could be utilized in any species with haploid material available (*e.g.*, social insects, haplontic plants and fungi) (Sandler *et al.* 2018; Yahav and Privman 2019).

### Improving de novo assembly transcriptomes with the haploid megagametophyte tissue

The most common strategy applied in *de novo* transcriptome assembly involves combining read data from several genotypes (individuals), developmental stages, and tissues of the target species. This is justified as a mean to capture most of the genes expressed under a variety of conditions and individuals. However, this also causes high levels of allelic redundancy and transcript fragmentation due to the high levels of heterozygosity and the genetic diversity across several genotypes (Ruttink *et al.* 2013). In conifers, this is the strategy routinely employed to generate *de novo* reference transcriptomes (López de Heredia and Vázquez-Poletti 2016), and it invariably leads to a large number of transcripts. Among the three strategies used here (Figure 1), combining all tissues and genotypes (strategy B) resulted in an assembly with the highest number of assembled contigs and predicted ORFs, but without necessarily being the most complete assembly (Table S3). On the other hand, the inclusion of the megagametophyte (haploid) during the *de novo* assembly as long read guidance (Figure 1C), increased the BUSCO completeness scores for both assemblers (Table 2). Contrary to *de novo* transcriptome hybrid assembly, where long reads (such as PacBio) are aligned to the short reads during the assembly (Fu *et al.* 2018), the long reads we used here from the megagametophyte (>500 bp) were not incorporated into the final assembly, and were only used to resolve isoforms during assembly. An additional strategy for generating more complete transcriptomes is the utilization of long read technologies (*e.g.*, Iso-Seq from PacBio or direct RNA from Nanopore), which allows to obtain full-length splice isoforms and several other post-transcriptional events (Zhao *et al.* 2019), with a few available approaches to cluster transcripts based on gene sequences (Marchet *et al.* 2019). The main benefit obtained from incorporating haploid tissue reads during the assembly stage is the increase of transcriptome completeness. The inclusion of haploid tissue did not considerably affect the number of transcripts or the ORFs predicted, regardless of the assembler used (Table S3), which were still considerably larger than the estimated number of genes in conifer genomes (Nystedt *et al.* 2013; Zimin *et al.* 2014; Stevens *et al.* 2016; Neale *et al.* 2017).

### Levels of paralog sequence collapse in the assembled transcriptomes and the effect of redundancy reducing strategies

In conifers, CD-HIT clustering is the most common strategy used to reduce allelic redundancy, along with CAP3, with similar levels of effectiveness using either strategy (Yang and Smith 2013). In contrast, generation of gene-based consensus transcript using isoform alignment (Lace) has been rarely applied in conifer trees (Ueno *et al.* 2018). The CD-HIT algorithm was originally designed to reduce large protein data sets into representative sequences based on homology (Li *et al.* 2001), not to reduce redundancy in transcriptomes. In our study, CD-HIT reduced the number of transcripts and predicted ORFs more than the generation of SuperTranscripts with Lace alone (Table S4). Both Lace and CD-HIT reduced the percentage of duplicated genes in the BUSCO set to about 5% of the genes (Figure 2). As a side effect, applying these clustering steps reduce the BUSCO completeness scores of the transcriptomes by nearly 20% in *P. sylvestris* (Table S4). In addition, Lace resulted in the inclusion of duplicated exons on the generated sequences, resulting in the generation of artificial mosaic transcripts. We identified a total of 44,535 mosaics on this assembly before applying the CD-HIT clustering. Contrary to CD-HIT and Lace, the OGA approach was less successful in the reduction of false heterozygous calls. For OGA assemblies, the observed heterozygosity was 25% higher than the expected heterozygosity, indicating a higher number of false heterozygous calls (Table 2). This was mainly due to the lack of completeness of the *P. taeda* and *P. lambertiana* reference genome annotations. As better and more complete reference genomes become available in conifers, this approach might be an effective strategy to reduce allelic redundancy, PSC and paralog read mapping.

### Effect of allelic redundancy on the development of exome capture probes and strategies to mitigate its effect

**Figure 3:**
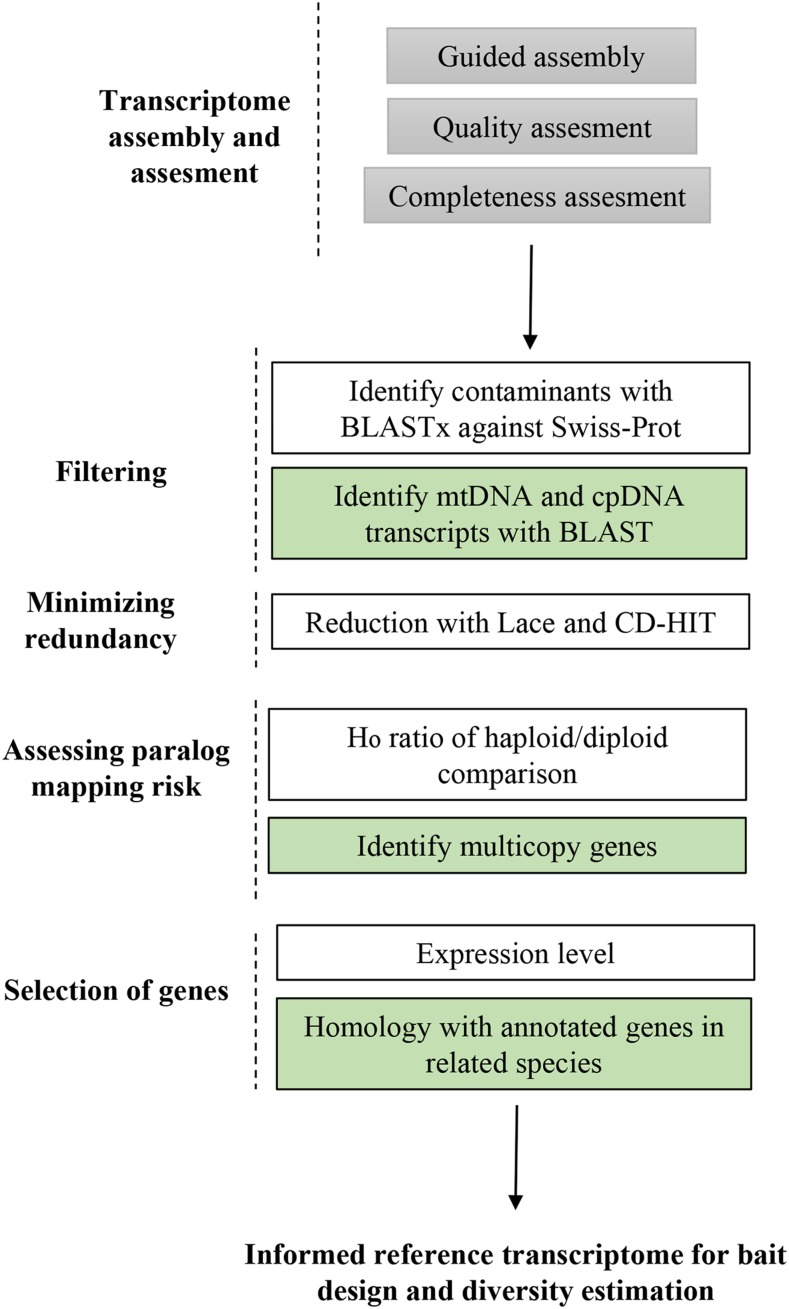
Strategies to employ the availability of haploid tissue in conifers for *de novo* transcriptome assembly and their application to assess the amount of allelic redundancy and paralog sequence collapse (PSC). Green boxes indicate additional steps recommended when additional genomic resources are available from a related species.

Appropriate selection of target genes is a crucial step for an effective target exome capture experiment, as the presence of paralog sequences, misassembled regions, mosaics, cpDNA and mtDNA decrease the efficiency of the recovery of the captured regions. When the design is entirely based on an assembled transcriptome, the most important step is to select the most appropriate contigs for bait design. For those references obtained with programs that group assembled contigs by graph component (multiple isoforms) such as Trinity, a representative sequence must be selected for bait design. This is usually accomplished by selecting the isoform with a minimum length (Suren *et al.* 2016), or combining information from isoform size and their expression level (Yang and Smith 2013). Based on our estimates of PSC, collapsing paralogs with Lace and CD-HIT after a haploid-guided assembly is a reasonable strategy to select suitable candidate genes. However, additional considerations should be added to further select the most suitable regions for bait design (Figure 3). Additional steps must include the removal of contaminant regions (Howe *et al.* 2013), genes from the organelle genomes (Syring *et al.* 2016), exclusion of potential mosaics produced by Lace, and the exclusion of transcripts with low level of expression. The exclusion of these regions will increase the efficiency in exome capture experiments, as well as of more accurate estimates of genetic diversity in population genetic analyses. In our case, 0.4% of the transcripts had a significant hit to chloroplast or mitochondrial *Pinus* genomes in the TRINITY_Lace_ assembly. Excluding genes with low levels of expression (cut-off of >10 TPM) reduced the number of genes (including their isoforms) to 108,860 after the TRINITY_guided_ assembly (Figure S5B). These low expressed genes are probably enriched with assembly artifacts and therefore not desirable in the bait design. For future reference and to help exome capture bait design of *P. sylvestris*, information on copy number, organelle genome match, isoform number, expression, putative contaminants and mosaics at gene level for Trinity_Lace_ gene level assembly is provided in Data S1.

One aspect that has been rarely explored in the assembled transcriptomes of conifers is the proportion of chimaeras, which has been estimated between 4–9% in the assembled transcriptomes evaluated so far (Ueno *et al.* 2018). There are currently available strategies to identify chimeras in assembled transcriptomes (*e.g.*, Yang and Smith 2013), but these rely on the quality of annotated proteomes from closely related taxa. Thus, how effective this strategy is in conifers remains to be determined. Finally, the intron-exon boundaries of the selected genes can be inferred using annotated genome references of related species, and excluded from the target regions of the bait design, as it has been shown to improve capture efficiency due to the hybridization on the probe-exon regions (Neves *et al.* 2013; Suren *et al.* 2016). However, the success of predicting these boundaries rely on the quality of available reference genomes from closely related taxa or low coverage shotgun sequencing of the target species. Depending on the availability of genomic resources from closely related taxa, we suggest several tailored steps that can be integrated to select suitable target genes for bait design (Figure 3).

### Influence of transcriptome assembly strategies on estimates of genetic diversity

The use of transcriptomes as a reference for obtaining SNPs and estimating genetic diversity is becoming a common strategy for non-model species that lack a reference genome (Gayral *et al.* 2013; Romiguier *et al.* 2014; Xu *et al.* 2016; Yan *et al.* 2017). However, several factors can introduce errors, and careful assessment of the assembled transcriptome is required before employing this downstream application. These include the presence of alleles, isoforms, and paralogs on the assembled transcriptome contigs. For instance, allelic redundancy allows reads to map to their respective allele without mismatches, and because variant calling algorithms can report no polymorphisms, this will cause an underestimation of genetic diversity. On the other hand, too greedy clustering of transcripts will result in PSC, the collapse of paralogous sequences and the removal of some of these from the reference. As a result, reads derived from the genes that were removed from the reference may map onto the remaining sequence and variant calling algorithms will report nucleotide substitutions that differentiate the paralogous genes as SNPs (Gayral *et al.* 2013).

Overall, the H_e_ per nucleotide for the assembled transcriptomes we assessed was lower than expected based on previous studies (Grivet *et al.* 2017), ranging from 1.2 × 10^−4^ to 8.3 × 10^−4^ (Table 2), an order of magnitude lower than previously reported in *P. sylvestris* (Pyhäjärvi *et al.* 2011). The minimum required read depth and missing data threshold we applied to our data set were relatively lenient, which has probably increased the number of callable sites relative to variant calls. In addition, allele-specific expression can also reduce the observed genetic diversity measured using RNA-seq data. Note that in this study the measures of diversity among haploid and diploid samples were used to evaluate different assemblies and were based on relative levels of heterozygosity. For precise estimates of genetic diversity, datasets with reads originating from DNA, deeper sequencing, more stringent filters and larger number of individuals should be used. We additionally suggest careful analysis of genetic diversity from multi-copy genes as they are especially prone to paralog mapping. Our estimation of heterozygosity on the haploid tissues were higher for multi-copy than single-copy genes (Figure S8), and the exclusion of multi-copy genes will decrease false SNPs in downstream analysis. This approach, however, comes with a caveat of possible bias resulting from ignoring fast evolving gene families from the analysis (Figure 3).

In conclusion, we found that the use of individual assemblies obtained from the haploid tissue as a guide improves *de novo* assembly and can be also be employed to assess the levels of paralogy; thus, the availability of haploid tissue could be also exploited on other organisms that lack available genome references. Strategies employed to decrease redundancy cause paralog sequence collapse and the reduction of transcriptome completeness. We suggest that collapsing paralogs with Lace and CD-HIT after a haploid-guided assembly is a reasonable strategy to generate reference transcriptomes with reduced levels of paralogy. Precautions should be taken to inspect the output from Lace, as we found that it sometimes produces mosaic transcripts. Elimination of lowly expressed genes, contaminants, and multi-copy genes will decrease false SNPs in downstream analysis. The reference transcriptomes obtained using these strategies should provide less biased estimates on population genetic analyses, as well as to select more suitable regions for target enrichment.
